# Neutrophil-to-apolipoprotein A1 ratio as a novel biomarker for prognosis in anti-NMDAR encephalitis: a retrospective cohort analysis

**DOI:** 10.3389/fneur.2026.1725493

**Published:** 2026-01-28

**Authors:** Jinwei Zhang, Ling Ling, Lei Xiang, Zhiying Wang, Youming Li, Wei Yue

**Affiliations:** Department of Neurology, Huanhu Hospital Affiliated to Tianjin Medical University, Tianjin, China

**Keywords:** anti-N-methyl-D-aspartate receptor encephalitis, neuroinflammation, neutrophil-to-apolipoprotein A1 ratio, prognosis, recurrence

## Abstract

**Objective:**

To investigate the correlation between the neutrophil-to-apolipoprotein A1 ratio (NAR) and disease severity, long-term prognosis, and risk of relapse in patients with anti-N-methyl-D-aspartate receptor (anti-NMDAR) encephalitis.

**Methods:**

This study included 125 patients with anti-NMDAR encephalitis as a retrospective cohort. Baseline clinical, laboratory, and imaging data was collected. Spearman’s correlation analysis was used to evaluate correlations between NAR, disease severity, and C-reactive protein (CRP) levels. Logistic regression and Cox proportional hazards models were used to analyze independent associations between NAR and poor prognosis and recurrence, respectively. The predictive performance of NAR was evaluated using receiver operating characteristic (ROC) curves. Mediation analysis was used to explore potential pathways of action. Sensitivity and subgroup analyses were performed to verify the reliability of the results.

**Results:**

The final modified Rankin’s score (mRS) score and recurrence rate were significantly higher in the high-NAR group than in the low-NAR group (both *p* < 0.05). NAR significantly and positively correlated with the initial mRS score (*r* = 0.308, *p* < 0.001) and CRP level (*r* = 0.486, *p* < 0.001). Multivariate analysis showed that NAR was an independent risk factor for poor prognosis (OR = 1.19, 95% confidence interval (CI): 1.02–1.38, *p* = 0.026) and recurrence (Hazard ratio (HR) = 1.13, 95% CI: 1.02–1.24, *p* = 0.017). ROC curve analysis showed that the area under the curve (AUC) for predicting poor prognosis with NAR was 0.724, the optimal cutoff value was 10.34, and the specificity was 92.2%. Mediation analysis showed that disease severity partially mediated the relationship between NAR and prognosis (effect rate, 41.7%).

**Conclusion:**

NAR is an independent predictor of poor disease prognosis and risk of recurrence in patients with anti-NMDAR encephalitis. Its high specificity helps identify high-risk patients early and accurately, giving this biomarker long-term prognostic value.

## 1 Introduction

Anti-N-methyl-D-aspartate receptor (anti-NMDAR) encephalitis is one of the most common types of autoimmune encephalitis (1). It is more common in young people, with a relatively higher proportion of female patients (1). The clinical manifestations are complex and diverse, such as mental and behavioral abnormalities, epileptic seizures, and consciousness disorders. It can even be life-threatening in severe cases (1). Although patient prognosis has improved overall with the widespread use of immunotherapy, some patients still face the risk of severe neurological deficits or disease recurrence (2). At present, there are no biological indicators that can identify high-risk patients early, objectively, and accurately, which greatly limits the scope of prognostic evaluation and individualized treatment strategies.

In recent years, neuroinflammation has been shown to be the link between the onset and progression of anti-NMDAR encephalitis (3). Neutrophils are key effector cells of the innate immune system. Research has shown that neutrophils not only participate in systemic inflammatory responses but may also directly exacerbate blood–brain barrier disruption and neuronal damage; the mechanisms include central nervous system infiltration and the release of neutrophil extracellular traps (NETs) (3, 4). On the other hand, there are complex interactions between lipid metabolism and immune inflammation. Apolipoprotein A1 (ApoA-1), the main functional component of high-density lipoproteins, is responsible for cholesterol reverse transport (5). Reportedly, it also has a strong anti-inflammatory, antioxidant, and endothelial protective properties (5). Reduced apoA-1 levels are closely related to the severity and poor prognosis of various neuroimmune and neurological diseases (6).

However, a single inflammatory or lipid indicator may not fully reflect the complex pathophysiological state of anti-NMDAR encephalitis. The neutrophil-to-apolipoprotein A1 ratio (NAR) is an emerging composite marker that encompasses both proinflammatory driving factors and endogenous protective mechanisms. According to recent studies, NAR has excellent prognostic value in cardiovascular diseases (7), malignant tumors (8), and cerebrovascular diseases (9). However, the clinical significance and relevance of NAR in anti-NMDAR encephalitis remain currently unclear.

This study aimed to systematically explore the correlation between NAR and disease severity, long-term prognosis, and recurrence risk in patients with anti-NMDAR encephalitis via a retrospective cohort analysis. This may help identify new biomarkers and a theoretical basis for the risk stratification and clinical management of patients.

## 2 Materials and methods

### 2.1 Patients and selection criteria

**Figure 1:**
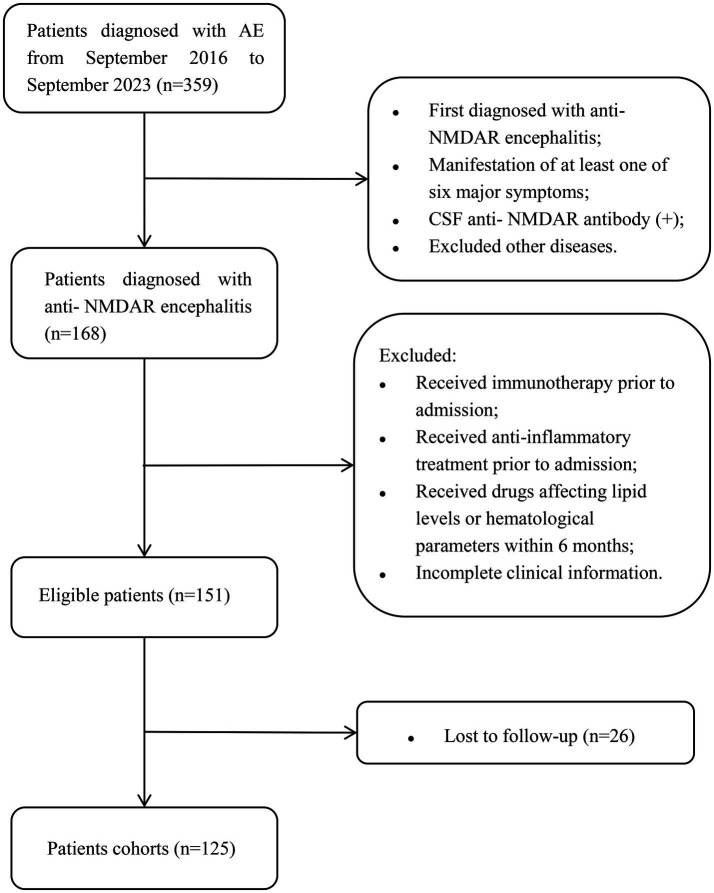
Flowchart of patient selection. AE, autoimmune encephalitis; anti-NMDAR, anti-N-methyl-D-aspartate receptor; CSF, cerebrospinal fluid.

This was a retrospective, observational, and single-center cohort study. We continuously screened patients with autoimmune encephalitis who were hospitalized at Tianjin Huanhu Hospital between September 2016 and September 2023. Inclusion criteria included: (1) Patients diagnosed with anti-NMDAR encephalitis for the first time according to the Graus and Dalmau criteria (10), manifesting at least one of the six following symptoms (a) abnormal mental behavior or cognitive impairment; (b) speech disorders; (c) epileptic seizures; (d) movement disorders, or involuntary movements; (e) reduced level of consciousness; (f) autonomic dysfunction or central hypoventilation; (2) Testing positive for anti-NMDAR antibody in cerebrospinal fluid (CSF); and (3) Exclusion of other causes. Exclusion criteria were as follows: (1) Treatment with immunotherapy, such as steroids, immunosuppressants, immunoglobulins, and serum replacement before admission; (2) Treatment with either anti-inflammatory medications carried out within 10 days before admission (a list of medications considered anti-inflammatory were included in Supplementary Table 1), or lipid-lowering treatments carried out within 6 months before admission, or medications that affected WBC (white blood cell) count and blood lipid levels within 6 months before admission; and (3) Incomplete clinical data or loss to follow-up. Loss to follow-up was defined as the inability to contact the patient through all existing contact methods (patient and emergency contact phone numbers). Throughout the entire follow-up period of the study, no patients died. In total, 125 patients with anti-NMDAR encephalitis were included in the final analysis (Figure 1). This study was approved by the Ethics Review Committee of Tianjin Huanhu Hospital (approval number: 202410281325000227905). Written informed consent to participate in this study was provided by the participants’ legal guardian/next of kin.

### 2.2 Clinical data collection

By reviewing the electronic medical records, two neurologists who were blinded to the research outcome independently collected data on demographics, age, and gender. Clinical characteristics included prodromal symptoms; core clinical manifestations such as mental and behavioral abnormalities, seizures, and consciousness disorders; and complications such as hypertension, diabetes, autoimmune diseases, and tumors. The treatment plan consisted of a choice between whether to receive first-line immunotherapy including corticosteroids, intravenous immunoglobulin, or plasma exchange; second-line immunotherapy, including rituximab or cyclophosphamide; or long-term immune maintenance therapy. Laboratory indicators collected included baseline blood test results of patients within 24 h of admission and before receiving immunotherapy, including routine blood tests, such as blood lipids, ApoA1, apolipoprotein B (ApoB), and C-reactive protein (CRP). NAR was calculated using the formula neutrophil count/ApoA1. Auxiliary examination included an assessment of CSF (white cell count, protein, glucose, chloride levels, and immune function) and MRI of the head.

### 2.3 Assessment of disease severity

The modified Rankin Scale (mRS) was used to assess the degree of neurological deficits in patients upon admission, defined as the initial mRS score (11). Scoring was independently conducted by two senior neurologists based on medical records at the time of admission. The mRS score ranged from 0 (asymptomatic) to 6 (death), with higher scores indicating more severe functional impairment.

### 2.4 Follow-up and outcomes

Follow-up was conducted via outpatient follow-ups or telephone interviews. Telephone interviews were conducted independently by two senior neurologists blinded to patients’ baseline information, using a structured mRS assessment questionnaire. Patients’ family members were invited to be present to assist in confirming the information. The main outcome was prognosis, while the mRS score at 12 months after onset was used as the final mRS score. Poor prognosis was defined as a final mRS score >2 points (12). The secondary outcome was recurrence, defined as the appearance of new or aggravated neurological symptoms after the initial treatment had improved or stabilized, when other causes such as infection had been ruled out (2). Recurrence time was defined as the time interval from the first onset to occurrence of the first recurrent event. For non-recurrent patients, the follow-up time was calculated until the last follow-up date. The follow-up deadline was September 30, 2025. This date ensured all patients have a common endpoint for observing the secondary outcome.

### 2.5 Statistical analyses

All statistical analyses in this study were conducted using IBM SPSS Statistics 26.0 (IBM Corp., Armonk, N. Y., USA) and GraphPad prism 8.0 (San Diego, CA, USA). The 125 patients included in the final analysis constituted a complete dataset, and all variables had no missing values. Based on the median NAR (5.70), all patients were divided into two groups (9, 13). The Kolmogorov–Smirnov test was used for normality testing. Quantitative data that conformed to a normal distribution were expressed as mean ± standard deviation (mean ± SD), and t-test was used for inter-group comparisons. Independent sample t-test was used for inter-group comparisons. Measurement data that did not conform to a normal distribution were represented by the median (interquartile range) [M (IQR)], and Mann–Whitney U test was used for inter-group comparisons. Count data were expressed as the number of cases (percentage) [*n* (%)], and comparisons between groups were performed using the chi-squared test or Fisher’s exact test. Spearman’s correlation analysis was used to evaluate the correlation between continuous variables such as NAR, neutrophils, disease severity (initial mRS), and CRP levels. To explore the independent predictive value of NAR for poor prognosis, we first conducted univariate logistic regression analysis and included variables with *p* < 0.05 in the multivariate logistic regression model to calculate the odds ratio (OR) and its 95% confidence interval (CI). To explore the independent predictive value of NAR for recurrence risk, we used univariate and multivariate Cox proportional hazards regression models to calculate hazard ratios (HR) and their 95% CI. We constructed two adjustment models to validate the robustness of the results. Model I was adjusted for basic demographic and clinical factors such as age, gender, and initial mRS. Model II was further adjusted for potential confounding factors, such as comorbidities and CRP. The potential role of the initial mRS score in the relationship between NAR and prognosis was examined using mediation analysis, while the 95% CI of indirect effects was calculated using the bootstrap method. The predictive power of the NAR was evaluated using a receiver operating characteristic (ROC) curve, and the area under the curve (AUC) was calculated. The optimal cut-off value for predicting prognosis was determined based on the maximum value of Youden’s index. Sensitivity and subgroup analyses were conducted to verify the reliability of the primary results. All statistical analyses were conducted using a two-sided test, and statistical significance was set at *p* < 0.05.

## 3 Results

### 3.1 Baseline clinical characteristics of patients

This study included 125 patients with anti-NMDAR encephalitis. As shown in Table 1, the median age of the entire study cohort was 31 (21–43) years, with adult patients (≥18 years old) accounting for 86.4% (104/120) and female patients accounting for 52.8% (63/120 patients). A total of 68 patients (54.4%) experienced prodromal symptoms in the early stages of the disease, including headache, fever, and vomiting.

**Table 1:** Comparison of clinical characteristics in patients with anti-NMDAR encephalitis stratified by median NAR.

Clinical characteristics	Total (*n* = 125)	NAR ≤ 5.70 (*n* = 63)	NAR > 5.70 (*n* = 62)	*p*
Demographic characteristics				
Age at onset, years, median (IQR)	31.00 (21.00–43.00)	32.00 (20.00–44.00)	31.00 (23.75–42.25)	0.671
Adults, ≥18, *n* (%)	108 (86.4)	53 (84.1)	55 (88.7)	0.455
Gender, female, *n* (%)	66 (52.8)	36 (57.1)	30 (48.3)	0.327
Personal history, *n* (%)				
Drinking	22 (17.5)	8 (12.6)	14 (22.5)	0.147
Smoking	24 (19.2)	13 (20.6)	11 (17.7)	0.681
Medical history, *n* (%)				
Hypertension	13 (10.4)	8 (12.6)	5 (8.0)	0.396
Diabetes	2 (1.6)	2 (3.1)	0 (0.0)	0.496
Autoimmune diseases	7 (5.6)	5 (7.9)	2 (3.2)	0.449
Tumor	9 (7.2)	4 (6.3)	5 (8.0)	0.980
Clinical manifestation, *n* (%)				
Prodrome	68 (54.4)	30 (47.6)	38 (61.2)	0.125
Consciousness disorders	41 (32.8)	19 (30.1)	22 (35.4)	0.526
Cognitive dysfunction	70 (59.6%)	39 (61.9)	31 (50.0)	0.180
Mental and behavioral abnormalities	98 (78.4)	48 (76.1)	50 (80.6)	0.545
Hallucination	22 (17.5)	13 (20.6)	9 (14.5)	0.369
Seizures	60 (48.0)	20 (31.7)	40 (64.5)	<0.001*
Speech dysfunction	64 (51.2)	32 (50.7)	32 (51.6)	0.927
Movement disorders	23 (18.4)	9 (14.2)	14 (22.5)	0.231
Involuntary facial movements	29 (23.2)	11 (17.5)	18 (29.0)	0.125
Involuntary limb movements	30 (24.0)	13 (20.6)	17 (27.4)	0.375
Autonomic dysfunction	33 (26.4)	13 (20.6)	20 (32.2)	0.140
Sleep dysfunction	46 (36.8)	24 (38.0)	22 (35.4)	0.762
Therapy regimens, *n* (%)				
First line immunotherapies	117 (93.6)	58 (92.0)	59 (95.1)	0.732
Second line immunotherapies	7 (5.6)	2 (3.1)	5 (8.0)	0.424
Long term immunotherapies	12 (9.6)	8 (12.6)	4 (6.4)	0.378
Follow-up time, median (IQR)	39.17 (29.02–53.64)	38.77 (28.53–53.00)	40.72 (32.28–56.69)	0.540
Final mRS, median (IQR)	2 (1–2)	1 (0–2)	2 (1–2)	<0.001*
Relapse, *n* (%)	23 (18.4)	6 (9.5)	17 (27.4)	0.010*

Continuous variables were presented as mean ± SD or median (IQR = 25th–75th percentile), and categorical variables were described as percentages (%).

anti-NMDAR, anti-N-methyl-d-aspartate receptor; NAR, neutrophil-to-apolipoprotein A1 ratio, mRS, modified Rankin scale. *p < 0.05.

Abnormal mental behavior (78.4%, 98/125 patients) and cognitive impairment (59.6%, 70/125 patients) were the most common clinical manifestations. Most patients (93.6%, 117/125 patients) received first-line immunotherapy (glucocorticoids, intravenous immunoglobulin, or plasma exchange), whereas only 5.6% (7/125 patients) and 9.6% (12/125 patients) received second-line immunotherapy (rituximab or cyclophosphamide) or long-term immune maintenance therapy (mycophenolate mofetil or azathioprine), respectively. The median follow-up time was 39.17 (29.02–53.64) months. At the last follow-up, the median final mRS score of the patients was 2 (1–2) points, and the overall recurrence rate of the cohort was 18.4% (23/125 patients).

Based on the median NAR (5.70), all patients were divided into a low-NAR group (≤5.70) and a high-NAR group (>5.70). There were no statistically significant differences between the two groups in terms of personal history, medical history, or most clinical manifestations (all *p* > 0.05). However, the proportion of epileptic seizures was significantly higher in the high-NAR group than in the low-NAR group (64.5% vs. 31.7%, *p* < 0.001). There were no significant differences in the first-, second-, and long-term immunotherapy regimens between the two groups (*p* > 0.05). Notably, the high-NAR group showed poorer clinical outcomes with significantly higher final mRS scores (*p* < 0.001) and a significantly higher recurrence relative to the low-NAR group (*p* = 0.010).

### 3.2 Laboratory and imaging examination results

A comparison of the laboratory and imaging results between the two groups of patients is presented in Table 2. 42.4% (53/125) of patients had positive serum antibodies. Compared to the low-NAR group, patients in the high-NAR group showed a higher state of systemic inflammation, with significantly increased WBC, neutrophil, and monocyte counts, along with higher CRP levels (all *p* < 0.001). Further, HDL-C and ApoA1 levels in the high-NAR group were significantly higher than those in the low-NAR group (both *p* < 0.001).

**Table 2:** Laboratory test and imaging results in patients with anti-NMDAR encephalitis stratified by median NAR.

Results	Total (*n* = 125)	NAR ≤ 5.70 (*n* = 63)	NAR > 5.70 (*n* = 62)	*p*
Laboratory test results				
Serum, median (IQR)				
WCC, *10^9^/L	8.80 (6.67–11.43)	6.8 (5.4–8.3)	11.43 (9.45–13.18)	<0.001*
RBC, *10^12^/L	4.24 (3.93–4.69)	4.25 (3.82–4.67)	4.24 (4.02–4.76)	0.379
Hb, g/L	4.28 ± 0.56	4.22 ± 0.51	4.33 ± 0.61	0.279
PLT, *10^9^/L	129.54 ± 18.45	127.44 ± 16.93	131.66 ± 19.78	0.203
Monocytes, ×10^9^/L	255.62 ± 78.20	251.76 ± 79.94	259.53 ± 76.85	0.581
Lymphocytes, ×10^9^/L	1.40 (1.01–2.03)	1.51 (1.09–2.26)	1.33 (0.90–1.59)	0.009*
Neutrophil, ×10^9^/L	6.58 (4.40–9.08)	4.42 (3.53–5.61)	9.08 (7.38–11.04)	<0.001*
TC, mmol/L	3.94 (3.37–3.94)	3.99 (3.41–4.79)	3.88 (3.35–4.39)	0.225
TG, mmol/L	1.00 (0.70–1.34)	1.05 (0.74–1.35)	0.93 (0.64–1.31)	0.270
HDL, mmol/L	1.08 (0.89–1.35)	1.11 (0.93–1.40)	1.03 (0.86–1.19)	0.049*
LDL, mmol/L	2.35 (1.93–2.96)	2.32 (2.07–3.07)	2.37 (1.85–2.86)	0.386
Apo-A1, g/L	1.09 (0.95–1.30)	1.22 (1.02–1.37)	0.98 (0.88–1.15)	<0.001*
Apo-B, g/L	0.86 (0.70–1.01)	0.78 (0.68–1.04)	0.88 (0.71–1.01)	0.400
CRP, mg/L	3.75 (1.30–8.99)	1.73 (0.76–5.00)	7.83 (1.98–17.32)	<0.001*
Serum anti-NMDAR IgG (+), *n* (%)	53 (42.4)	30 (47.6)	23 (37.0)	0.234
CSF, median (IQR)				
Pressure, mmH_2_O	170.00 (120.00–217.50)	160 (115–200)	170.00 (133.75–240.00)	0.127
WCC, *10^6^/L	14.00 (4.00–46.50)	8.00 (4.00–32.00)	23.00 (6.00–65.00)	0.011*
Protein, g/L	0.31 (0.21–0.46)	0.32 (0.21–0.46)	0.31 (0.21–0.50)	0.929
Glucose, mmol/L	3.29 (2.78–4.09)	3.45 (2.85–4.04)	3.19 (2.78–4.23)	0.305
Chloride, mmol/L	125.90 (123.80–128.00)	125.7 (123.1–128)	125.95 (123.88–128.03)	0.480
IgG, mg/L	39.90 (24.65–65.35)	41.80 (24.30–66.60)	37.85 (24.68–65.02)	0.724
Albumin, g/L	167.70 (124.50–290.90)	196.2 (124.5–325.5)	162.50 (110.70–278.13)	0.219
Q-IgG^a^	3.30 (2.15–5.97)	3.50 (2.14–5.29)	3.27 (2.15–6.03)	0.980
Q-Alb^b^	4.40 (2.93–7.21)	4.41 (2.98–7.84)	4.07 (2.81–6.95)	0.370
IgG index	0.73 (0.61–0.97)	0.68 (0.59–0.89)	0.78 (0.63–0.97)	0.067
Abnormal head-MRI, *n* (%)	75 (60.0)	42 (66.6)	33 (53.2)	0.125
Cerebral cortex/subcortical WM	63 (50.4)	37 (58.7)	26 (41.9)	0.060
Corpus callosum	2 (1.6)	1 (1.5)	1 (1.6)	0.748
Periventricular WM	23 (18.4)	14 (22.2)	9 (14.5)	0.266
Thalamus	13 (10.4)	6 (9.5)	7 (11.3)	0.746
Basal ganglia	8 (6.4)	4 (6.3)	4 (6.5)	1.000
Hippocampus	8 (6.4)	4 (6.3)	4 (6.5)	1.000
Insula	5 (4.0)	3 (4.7)	2 (3.2)	1.000
Cerebellum	3 (2.4)	2 (3.1)	1 (1.6)	1.000
Brainstem	7 (5.6)	5 (7.9)	2 (3.2)	0.449

Continuous variables were presented as mean ± SD or median (IQR = 25th–75th percentile), and categorical variables were described as percentages (%).

anti-NMDAR, anti-N-methyl-d-aspartate receptor; NAR, neutrophil-to-apolipoprotein A1 ratio, WCC, white cell count; RBC, red cell count; Hb, hemoglobin; PLT, platelets; TC, total cholesterol; TG, triglycerides; HDL, high density lipoprotein; LDL, low density lipoprotein; Apo-A1, apolipoprotein A1; Apo-B, apolipoprotein B; CRP, C-reactive protein; CSF, cerebrospinal fluid; MRI, magnetic resonance imaging; WM, white matter.

^a^Q-IgG is the ratio of CSF IgG to serum IgG; ^b^Q-Alb is the ratio of CSF albumin to serum albumin. *p < 0.05.

The WBC count in the CSF of the high-NAR group was significantly higher than that in the low-NAR group (*p* = 0.011), indicating more severe infiltration of inflammatory cells in the central nervous system (CNS). However, there were no statistically significant differences between the two groups in terms of initial CSF pressure, protein quantification, chloride and glucose levels, albumin quotient values reflecting blood–brain barrier integrity, IgG quotient values reflecting intrathecal synthesis, or the IgG index (all *p* > 0.05).

MRI of the head showed that 60.0% (75/125) of patients had abnormal signals. The most common distribution sites of these abnormal signals were the cerebral cortex/subcortical white matter (50.4%) and the periventricular white matter (18.4%). There was no significant difference in the rate of abnormal MRI signals and lesion distribution between the two groups (both *p* > 0.05).

### 3.3 Correlation between NAR, disease severity and systemic inflammation

Spearman’s correlation analysis showed (Supplementary Table 2; Supplementary Figure 1) that NAR showed significant positive correlated with disease severity (initial mRS score) (*r* = 0.308, *p* < 0.001). Furthermore, NAR showed a stronger significant positive correlation with the classic inflammatory marker CRP (*r* = 0.486, *p* < 0.001) (Supplementary Table 3).

Other indicators of lipid metabolism, including total cholesterol, triglycerides, LDL-C, HDL-C, and apolipoprotein B (apoB), did not significantly correlate with disease severity (initial mRS) or CRP levels (all *p* > 0.05).

### 3.4 Logistic regression analysis of poor prognosis

The results of univariate analysis (Table 3) showed that higher neutrophil count (OR = 1.20, 95% CI: 1.07–1.36, *p* = 0.003), NAR (OR = 1.23, 95% CI: 1.09–1.39, *p* = 0.001), and higher initial mRS score (OR = 1.41, 95% CI: 1.02–1.97, *p* = 0.041) were risk factors for poor prognosis. A higher lymphocyte count was observed as a protective factor (OR = 0.30, 95% CI: 0.13–0.73, *p* = 0.008).

**Table 3:** Univariate binary logistic regression analysis of poor outcome in patients with anti-NMDAR encephalitis.

Factor	Univariate analysis	
	OR (95% CI)	*p*
Age at onset	1.03 (0.99–1.06)	0.069
Gender, female	0.60 (0.22–1.42)	0.222
Hypertension	0.36 (0.04–2.93)	0.341
Autoimmune diseases	0.77 (0.09–6.74)	0.813
Tumor	0.57 (0.07–4.77)	0.600
Drinking	2.04 (0.69–6.00)	0.196
Smoking	1.77 (0.61–5.15)	0.294
Serum anti- NMDAR antibody (+)	1.16 (0.46–2.94)	0.750
Monocytes	1.10 (0.24–5.13)	0.903
Lymphocytes	0.30 (0.13–0.73)	0.008*
Neutrophil	1.20 (1.07–1.36)	0.003*
TC	1.27 (0.80–2.03)	0.315
TG	0.70 (0.33–1.49)	0.358
HDL	1.48 (0.33–6.58)	0.610
LDL	1.25 (0.71–2.20)	0.447
Apo-A1	0.14 (0.02–1.16)	0.068
Apo-B	3.84 (0.56–26.18)	0.169
CRP	1.02 (0.99–1.04)	0.157
NAR	1.23 (1.09–1.39)	0.001*
Abnormal head-MRI	0.49 (0.19–1.23)	0.130
Initial mRS	1.41 (1.02–1.97)	0.041*

anti-NMDAR, anti-N-methyl-d-aspartate receptor; OR, odds ratio; CI, confidence interval; TC, total cholesterol; TG, triglycerides; HDL, high density lipoprotein; LDL, low density lipoprotein; Apo-A1, apolipoprotein A1; Apo-B, apolipoprotein B; CRP, C-reactive protein; NAR, neutrophil-to-apolipoprotein A1 ratio; MRI, magnetic resonance imaging; mRS, modified Rankin scale. *p < 0.05.

To control for the influence of potential confounding factors, we constructed two multivariate adjustment models to further validate the independence of NAR. Model I was adjusted for basic clinical factors, such as age of onset, gender, and initial mRS score. Model II was further adjusted for comorbidities, such as autoimmune diseases, tumors, CRP, and lymphocyte counts. Both models showed that the association between NAR and poor prognosis was independent and significant (*p* < 0.05). Specifically, in Model II, for every 1 unit increase in NAR, the risk of poor prognosis increased by 19% (Table 4).

**Table 4:** Multivariate binary logistic regression analysis of poor outcome in patients with anti-NMDAR encephalitis.

Factor	Model I		Model II	
	OR (95% CI)	*p*	OR (95% CI)	*p*
Age at onset	1.03 (0.99–1.07)	0.086	1.03 (0.99–1.06)	0.179
Gender, female	0.69 (0.25–1.93)	0.480	0.62 (0.20–1.89)	0.403
NAR	1.22 (1.08–1.39)	0.002*	1.19 (1.02–1.38)	0.026*
Initial mRS	1.18 (0.81–1.71)	0.385	1.15 (0.78–1.07)	0.489
Autoimmune diseases	-	-	1.09 (0.08–15.38)	0.949
Tumor	-	-	0.87 (0.07–11.07)	0.914
CRP	-	-	1.00 (0.97–1.04)	0.791
Lymphocytes	-	-	0.44 (0.17–1.18)	0.102

anti-NMDAR, anti-N-methyl-d-aspartate receptor; OR, odds ratio; CI, confidence interval; CRP, C-reactive protein; NAR, neutrophil-to-apolipoprotein A1 ratio; mRS, modified Rankin scale. *p < 0.05.

### 3.5 Predictive value of NAR for poor disease prognosis

**Figure 2:**
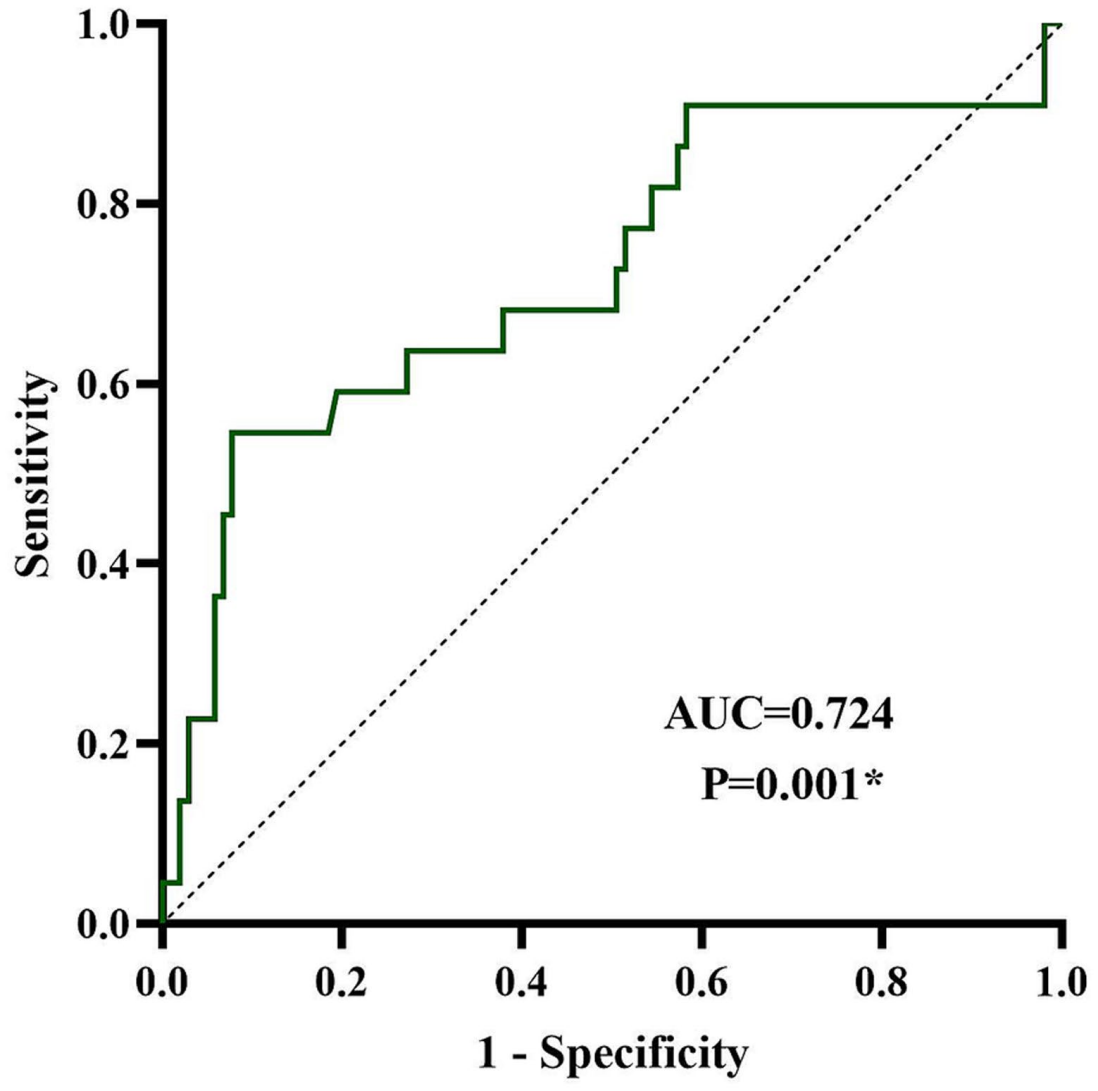
ROC curve analysis of the diagnostic value of NAR for disease prognosis in anti-NMDAR encephalitis. NAR, neutrophil-to-apolipoprotein A1 ratio; anti-NMDAR, anti-N-methyl-D-aspartate receptor. ^*^p < 0.05.

The analysis results showed that the area under the curve (AUC) for predicting poor prognosis with NAR was 0.724 (95% CI: 0.59–0.86), and its predictive value had significant statistical significance (*p* = 0.001). The optimal cutoff value was 10.34, with a specificity of 92.2% and a sensitivity of 54.5%. When the NAR of a patient was greater than 10.34, there was a high possibility of poor prognosis in the later stages of the disease (Figure 2).

### 3.6 The mediating effect of disease severity between NAR and poor prognosis

**Figure 3:**
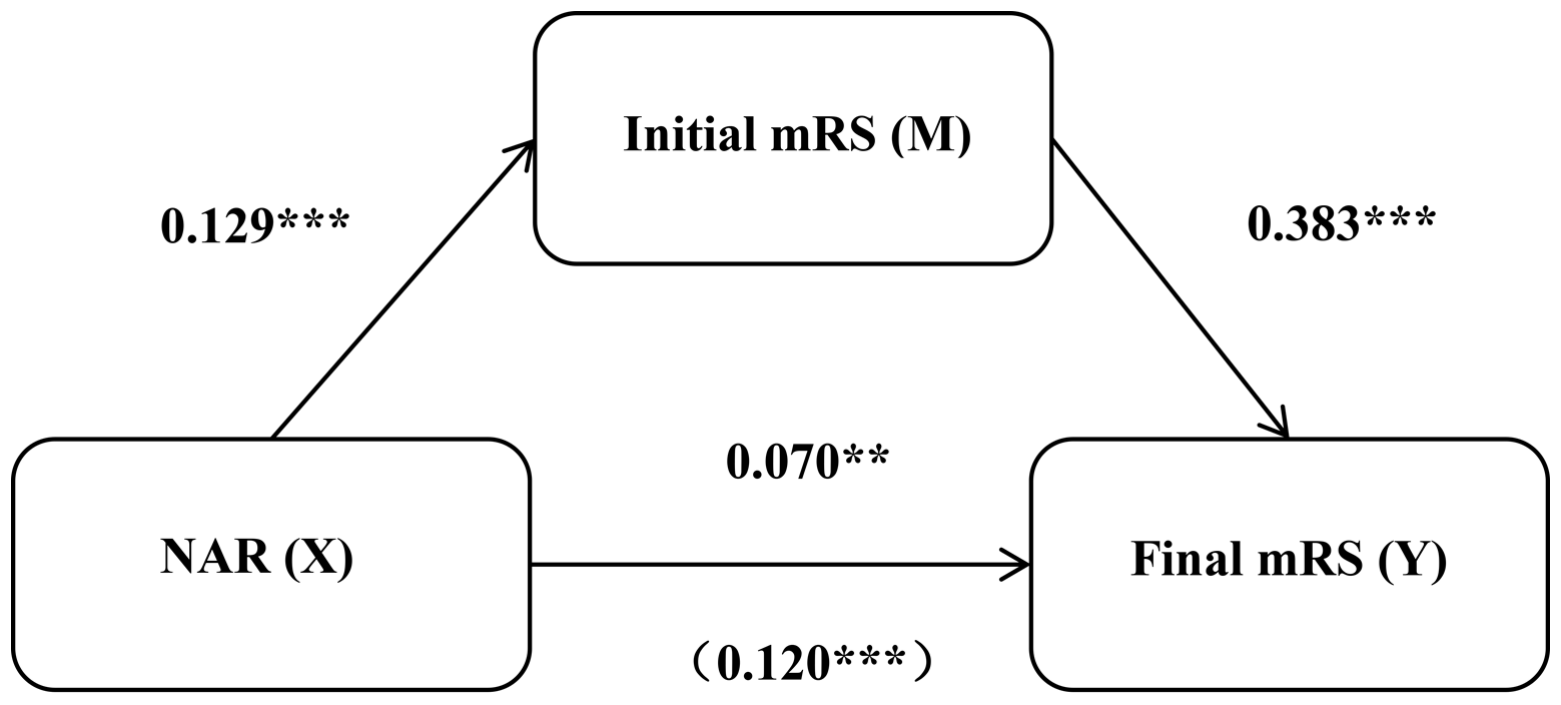
The mediating role of initial mRS between NAR and disease prognosis in anti-NMDAR encephalitis. mRS, Modified Rankin Scale; NAR, neutrophil-to-apolipoprotein A1 ratio; anti-NMDAR, anti-N-methyl-D-aspartate receptor. ***p < 0.001, **p < 0.01, *p < 0.05.

To investigate the potential pathways through which NAR affects prognosis, we used a mediation effect model to test the role of the initial mRS in NAR and poor prognosis. The results are shown in Table 5 and Figure 3. All regression paths reached a statistically significant level: NAR had a significant positive effect on the initial mRS (*β* = 0.129, *p* < 0.001). The initial mRS has a significant positive impact on prognosis score (*β* = 0.383, *p* < 0.001). NAR has a significant impact on prognosis score (*β* = 0.070, *p* = 0.003), with a direct effect of 0.07 (95% CI: 0.025–0.116), accounting for 58.3% of the total effect. After adding mediator variables, the impact of NAR on poor prognosis mediated by disease severity remained significant (*β* = 0.120, *p* < 0.001), with an indirect effect of 0.050 (95% CI: 0.027–0.079), accounting for 41.7% of the total effect. Disease severity plays a partial mediating role in the relationship between NAR and poor prognosis.

**Table 5:** The mediating role of initial mRS between NAR and poor outcome of anti-NMDAR encephalitis.

Effect type	Effect	SE	LLCI	ULCI
Total effect	0.120	0.025	0.070	0.170
Direct effect	0.070	0.023	0.025	0.116
Indirect effect	0.050	0.013	0.027	0.079

mRS, modified Rankin scale, anti-NMDAR, anti-N-methyl-d-aspartate receptor; NAR, neutrophil-to-apolipoprotein A1 ratio; SE, standard error; LLCI, lower level of confidence interval; ULCI, upper level of confidence interval.

### 3.7 Cox regression analysis of recurrence

**Figure 4:**
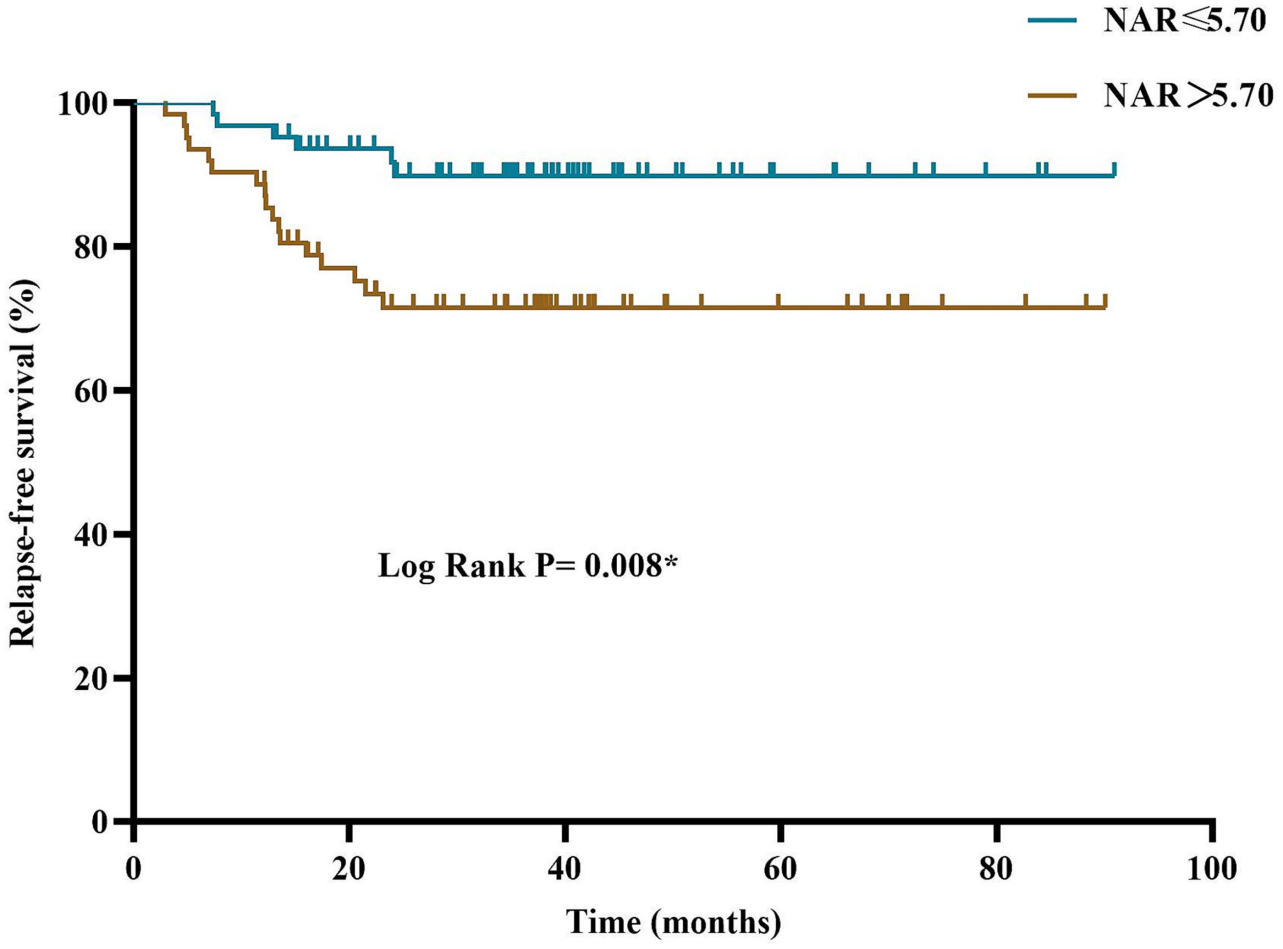
Kaplan–Meier curves categorized according to NAR. NAR, neutrophil-to-apolipoprotein A1 ratio. *p < 0.05.

The Kaplan–Meier curve showed that elevated NAR is a significant risk factor for long-term recurrence in patients with anti-NMDAR encephalitis (Figure 4). The cumulative recurrence rate was significantly higher in the high-NAR group than in the low-NAR group. The log-rank test showed that the difference between the two groups was statistically significant (*p* = 0.008). At the 12-month follow-up, the cumulative recurrence rate in the low-NAR group was 3.2% (2/63 patients), while in the high NAR group it was 11.3% (7/62 patients). At 24 months, the cumulative recurrence rate in the low-NAR group was 7.9% (5/63 patients), while that of the high-NAR group increased to 27.4% (17/62 patients) (Figure 4).

Univariate analysis (Table 6) showed that baseline neutrophil count (HR = 1.14, 95% CI: 1.05–1.25, *p* = 0.003), NAR (HR = 1.14, 95% CI: 1.05–1.24, *p* = 0.001), and initial mRS score (HR = 1.38, 95% CI: 1.03–18.6, *p* = 0.030) were significantly associated with a higher risk of recurrence during follow-up. To verify the independence of NAR, we constructed a multifactorial model (Table 7). Model I was adjusted for age at onset, gender, and initial mRS score, whereas Model II was further adjusted for concomitant autoimmune diseases, concomitant tumors, and CRP levels. The results showed that NAR remained an independent risk factor for recurrence risk (HR = 1.13, 95% CI: 1.02–1.24, *p* = 0.017). In Model II, for every 1 unit increase in the NAR, the patient’s risk of recurrence increased by 13% (Table 7).

**Table 6:** Univariate Cox analysis of relapse in patients with anti-NMDAR encephalitis.

Factor	Univariate analysis	
	HR (95% CI)	*p*
Age at onset	1.00 (0.97–1.02)	0.841
Gender, female	0.56 (0.24–1.30)	0.180
Hypertension	2.15 (0.73–6.32)	0.166
Autoimmune diseases	0.75 (0.10–5.59)	0.782
Tumor	0.61 (0.08–4.51)	0.627
Drinking	1.33 (0.49–3.58)	0.573
Smoking	2.00 (0.82–4.86)	0.127
Serum anti- NMDAR antibody (+)	1.57 (0.69–3.55)	0.283
Monocytes	2.63 (0.87–7.92)	0.087
Lymphocytes	1.07 (0.59–1.97)	0.816
Neutrophil	1.14 (1.05–1.25)	0.003*
TC	0.77 (0.48–1.23)	0.269
TG	0.97 (0.71–1.33)	0.859
HDL	1.06 (0.28–4.00)	0.930
LDL	0.76 (0.43–1.34)	0.346
Apo-A1	0.29 (0.05–1.77)	0.180
Apo-B	0.74 (0.12–4.43)	0.738
CRP	1.01 (0.99–1.03)	0.283
NAR	1.14 (1.05–1.24)	0.001*
Abnormal head-MRI	0.70 (0.31–1.60)	0.401
Initial mRS	1.38 (1.03–1.86)	0.030*

anti-NMDAR, anti-N-methyl-d-aspartate receptor; HR, hazard ratio; CI, confidence interval; TC, total cholesterol; TG, triglycerides; HDL, high density lipoprotein; LDL, low density lipoprotein; Apo-A1, apolipoprotein A1; Apo-B, apolipoprotein B; CRP, C-reactive protein; NAR, neutrophil-to-apolipoprotein A1 ratio; MRI, magnetic resonance imaging; mRS, modified Rankin scale. *p < 0.05.

**Table 7:** Multivariate Cox analysis of relapse in patients with anti-NMDAR encephalitis.

Factor	Model I		Model II	
	OR (95% CI)	*p*	OR (95% CI)	*p*
Age at onset	0.99 (0.96–1.02)	0.389	0.99 (0.96–1.02)	0.380
Gender, female	0.52 (0.22–1.25)	0.142	0.55 (0.22–1.37)	0.197
NAR	1.12 (1.03–1.22)	0.010*	1.13 (1.02–1.24)	0.017*
Initial mRS	1.29 (0.94–1.76)	0.114	1.30 (0.94–1.79)	0.109
Autoimmune diseases	-	-	1.73 (0.21–14.27)	0.612
Tumor	-	-	0.59 (0.07–5.16)	0.637
CRP			1.00 (0.98–1.03)	0.953

anti-NMDAR, anti-N-methyl-d-aspartate receptor; OR, odds ratio; CI, confidence interval; CRP, C-reactive protein; NAR, neutrophil-to-apolipoprotein A1 ratio; mRS, modified Rankin scale. *p < 0.05.

### 3.8 Sensitivity analysis of results after excluding combined autoimmune diseases

To verify the reliability of the above results and exclude potential interference from combined autoimmune diseases, we conducted a sensitivity analysis (Supplementary Tables 4, 5). The analysis showed that after excluding patients with concomitant autoimmune diseases, NAR still significantly associated with poor disease prognosis in both multiple logistic regression models (Models I and II), and the effect values were consistent with the analysis of the entire population (both *p* < 0.05). In both multivariate Cox regression models (models I and II), NAR also maintained its significance as an independent risk factor for recurrence (both *p* < 0.05). The sensitivity analysis indicated that the association between NAR and poor prognosis and recurrence risk was independent.

### 3.9 Subgroup analysis of NAR predictive value between different genders

To investigate whether gender affected the association between NAR and clinical outcomes, we conducted a subgroup analysis of the interaction tests (Supplementary Table 6). The analysis showed no statistically significant interaction between gender and NAR for poor disease prognosis and disease recurrence (*p* > 0.05); therefore, the predictive effect of NAR on poor prognosis and recurrence risk is consistent among patients of different genders with anti-NMDAR encephalitis.

## 4 Discussion

This study systematically explored the clinical significance of NAR in anti-NMDAR encephalitis for the first time via a retrospective cohort analysis of 125 patients. Our results indicate that NAR significantly correlates with the severity of acute phase of the disease and is an independent predictor of poor long-term functional prognosis and long-term recurrence risk. This association remained significant after adjusting for various confounding factors, including the traditional inflammatory marker CRP. The findings of this study provide a potential new biomarker for the prognostic evaluation of anti-NMDAR encephalitis, which is easily accessible and derived from routine blood tests.

Neutrophils are the most abundant WBCs in the human body. They are the key effector cells of the innate immune system and the first line of defense against infection and damage (14). They play a crucial role in clearing pathogens, protecting host tissues, and maintaining tissue homeostasis (15, 16). Numerous studies have confirmed that neutrophil-mediated inflammatory responses are involved in the progression of various autoimmune diseases such as myasthenia gravis (MG), multiple sclerosis (MS), systemic lupus erythematosus (SLE), and anti-NMDAR encephalitis (17–20). Early expansion and infiltration of circulating neutrophils have been observed both in models of experimental autoimmune encephalitis (EAE) and patients with MS (21, 22). In patients with neuromyelitis optica spectrum disorder (NMOSD), lesion severity increases with an increase in neutrophil count, and decreases with a reduction in neutrophil count (23). In this study, we found that neutrophil count not only significantly positively correlates with CRP levels and initial disease severity, but is also an independent risk factor for poor prognosis and recurrence of anti-NMDAR encephalitis. This discovery is similar to previous research findings in neuroimmune diseases such as MS and EAE, jointly demonstrating the important role of neutrophils in autoimmune CNS disorders.

The possible pathogenic mechanism of anti-NMDAR encephalitis begins in the preclinical stage of the disease, where inflammatory factors such as the pro-inflammatory cytokines IL-17A and TNF-*α* secreted by the key effector cells Th17 directly induce systemic upregulation of neutrophil granulocyte colony-stimulating factor (G-CSF) and ELR + CXC chemokines (24). In diseases such as NMOSD, neutrophil-associated CXC chemokines, such as CXCL1, CXCL5, and CXCL7, are significantly elevated in the CSF (25). These factors work together to drive the expansion and mobilization of neutrophils in the bone marrow, which accumulate in large quantities in the peripheral circulation (26). Subsequently, neutrophils are recruited and infiltrate the CNS. Activated neutrophils directly disrupt the integrity of the blood–brain barrier (BBB) and the blood-spinal cord barrier (BSCB). They exacerbate neuroinflammation by secreting substances such as neutrophil elastase, myeloperoxidase (MPO), reactive oxygen species (ROS), or the pro-inflammatory cytokine interleukin-1 *β* (IL-1 β) (27, 28). This may be an important mechanism that leads to worsening of neurological deficits and poor prognosis in patients with anti-NMDAR encephalitis. The formation of neutrophil extracellular traps (NETs) is a core component of the inflammatory response. Excessive or abnormal formation of NETs not only exacerbates autoimmune reactions (29) but also directly damages neurons and glial cells (30), activates the complement system, macrophages, and dendritic cells, amplifying local inflammation (31–33). Per previous research, the NETs marker citrullinated histone H3 (H3Cit) in patients with anti-NMDAR encephalitis positively correlates with levels of pro-inflammatory cytokines such as IL-6, IL-8, TNF-*α*, resulting in a vicious cycle of “cytokine triggered NET release, and NETs promoting inflammatory response expansion” (3, 34, 35). In addition, infiltrating neutrophils can directly interact with B- and T-cells by expressing molecules such as CD40L, B-cell activating factor (BAFF), and the protein A Proliferation-Inducing Ligand (APRIL), thereby regulating cellular and humoral immune responses (25). A prolonged lifespan caused by the inhibition of neutrophil apoptosis at the site of inflammation leads to persistent chronic inflammation, which is also an important cause of sustained tissue damage. The inflammatory microenvironment created by activated neutrophils may be closely related to long-term disease recurrence.

ApoA-1, an important anti-inflammatory protein (36), is the main structural and functional carrier of HDL-C. It is involved in cholesterol reverse transport and the clearance of excess cholesterol in peripheral tissues. Under acute inflammatory conditions, apoA-1 levels decrease significantly (5). Per previous studies, apoA-1 levels significantly correlate with poor prognosis and recurrence in patients with autoimmune encephalitis (AE) (6). In patients with anti-NMDAR encephalitis, serum apoA-1 level is reduced. Further, the apoB to apoA-1 ratio positively correlates with worse baseline conditions, longer hospital stays, and inflammatory markers CRP and erythrocyte sedimentation rate (ESR), suggesting that it can serve as a potential prognostic indicator (6, 37). In patients with MS, low apoA-1 levels associate with disease progression, more severe axonal injury, and higher levels of pro-inflammatory cytokines (38). In the EAE model, apoA-1 deficiency leads to more severe clinical manifestations (39). In addition, a negative correlation has been observed between apoA-1 levels and disease activity, neurological deficits, and recurrence risk in patients with NMOSD (40) and SLE (41, 42). A previous study also found a significant correlation between apoA-1 and CRP levels in patients with AE (43).

ApoA-1 exerts anti-inflammatory effects primarily by regulating immune cells (5). ApoA-1 can inhibit the inflammatory activity of monocytes and macrophage infiltration, and reduce the production of pro-inflammatory cytokines (39). ApoA-1 can regulate lipid raft cholesterol efflux, inhibit the interaction between dendritic cells (DCs) and natural killer cells (NK), and lead to a decrease in cytokines such as IFN-*γ* and IL-12 p70 (5). Although apoA-1 is not synthesized in the brain, it can penetrate the BBB and exert anti-inflammatory and neuroprotective effects in the CNS (38). It can inhibit excessive activation of microglia and block their pathological interactions with infiltrating T-cells (29). It also protects endothelial cells, maintains BBB integrity, and reduces neuroinflammation by inhibiting the expression of vascular cell adhesion molecule-1 (VCAM-1) mediated by NF-*κ* B (44). Additionally, apoA-1 plays an important role in reducing antioxidant stress. ApoA-1 can significantly reduce the oxidative modification of LDL-C by activating the complement system, directly neutralizing or clearing reactive oxygen species (ROS), and exerting anti-apoptotic effects (45). Recent studies have also revealed the potential value of apoA-1 in reducing ferroptosis and protecting neurons (36). In contrast to the significant pro-inflammatory effects of neutrophils, apoA1, which displays both anti-inflammatory and protective effects, showed more complex effects in this study. ApoA-1 levels showed a negative correlation with disease severity and CRP levels in patients with anti-NMDAR encephalitis, although no statistical significance was found. However, lower levels of apoA-1 had a moderate impact on poor prognosis and recurrence, again without statistical significance. ApoA-1 did not appear an independent significant predictor in this cohort. This may indicate that apoA-1 on its own may not be sufficient to represent its functional status in complex inflammatory environments during the acute and severe inflammation state of anti-NMDAR encephalitis, or that its protective effect may be masked by dominant strong pro-inflammatory responses.

Although apoA-1 itself was not predictive of disease severity, prognosis, or recurrence, the neutrophil to apoA-1 ratio, or NAR, showed strong predictive ability. Per previous studies, NAR significantly associates with poor prognosis in hepatocellular carcinoma (8), pancreatic cancer (46), acute heart failure (7), acute ischemic stroke (9) and other diseases. Unlike a single indicator, NAR combines “pro-inflammatory driving factors” with “endogenous protective mechanisms” to more comprehensively reflect the immune status in the body. ApoA-1 has a multilevel inhibitory effect on neutrophils. ApoA-1 can inhibit neutrophil production by reducing G-CSF production at the source (5, 46, 47); directly inhibit the activation, adhesion, diffusion, and migration ability of mature neutrophils by reducing the abundance of cell membrane lipid rafts (8); and inhibit the synthesis of interleukin-8 (IL8) to limit neutrophil recruitment (48).

The results of this study indicated a significant positive correlation between NAR and CRP, confirming that NAR can serve as an effective indicator of systemic inflammation in the body. Univariate and multivariate analyses showed that NAR was an independent risk factor for poor prognosis and long-term recurrence in patients with anti-NMDAR encephalitis. Of particular importance, the ROC curve analysis determined the optimal critical value for predicting prognosis with NAR to be 10.34, with a specificity of 92.2% and a sensitivity of 54.5%, indicating that when a patient’s NAR exceeds this level, the risk of poor prognosis is high. About half of low NAR individuals may have poor outcomes. The possible reason was that the prognosis of anti-NMDAR encephalitis was determined by multiple factors. NAR reflected a specific pathological pathway of “pro-inflammatory-anti-inflammatory imbalance.” For patients with low NAR but experienced poor prognosis, their poor outcomes may be mainly influenced by other mechanisms, such as non-inflammatory factors like high initial mRS, delayed treatment, or complications. This discovery has clear clinical value and can help accurately identify high-risk populations that require enhanced intervention in the early stages of the disease. Furthermore, the mediation analysis showed that disease severity partially mediated the impact of NAR on prognosis, suggesting that a systemic inflammatory state represented by a high NAR can worsen long-term functional outcomes by exacerbating acute CNS damage. In addition, the robustness of this study was validated through multidimensional analysis, which showed that the predictive role of NAR remained unchanged after excluding patients with concomitant autoimmune diseases. Subgroup analysis also did not find a significant interaction between gender and NAR, indicating that the predictive value of this indicator is consistent across different gender groups. Therefore, for patients with a significantly elevated NAR, even if their acute-phase treatment response is good, clinical attention should be paid to their high risk of recurrence, considering extending the consolidation treatment period and strengthening long-term follow-up to achieve more effective full-course disease management. From a therapeutic perspective, early intervention to reduce neutrophil levels and control the inflammatory status may become an important strategy for improving the clinical outcomes of anti-NMDAR encephalitis. Simultaneously, targeted therapy for apoA-1 has also shown broad prospects–such as the apoA-1 mimetic peptide–which is in the preliminary research stage and has shown significant anti-inflammatory and endothelial function-improving effects in animal models (49). In the future, a dual strategy of comprehensively regulating pro-inflammatory factors and enhancing protective mechanisms is expected to provide more effective treatment options for patients, thereby improving the long-term prognosis and reducing the risk of recurrence.

This study has some limitations. First, this was a single-center retrospective study. Patient selection and data collection may have been affected by an inherent bias, and the treatments could not be fully controlled. We did not collect data such as neuropsychological tests and cognitive function tests, which could affect a more comprehensive interpretation of disease prognosis. Second, the definition of recurrence was based on clinical criteria without systematic biomarker verification (such as CSF antibody titers), which was a limitation of the retrospective design. Third, we mainly focused on blood biomarkers and lacked data on inflammatory factors in the CSF (such as IL-6 and IL-17) during the same period. Thus, we were unable to fully reveal the direct association between NAR and CNS-specific inflammation. Finally, although we adjusted for known confounding factors using statistical models, some uncontrollable confounding factors, such as lifestyle habits and diet, may also have affected the results.

In summary, this study systematically confirmed for the first time that NAR, as a novel and readily available composite inflammatory marker, is an independent and robust predictor of adverse functional prognosis and long-term recurrence risk in patients with anti-NMDAR encephalitis. NAR integrates “pro-inflammatory” and “anti-inflammatory” pathophysiological processes, enabling a more comprehensive assessment of the overall inflammatory imbalance in patients. This study not only provides important evidence for risk stratification of anti-NMDAR encephalitis, but also offers new ideas for improving disease progression and long-term prognosis through early intervention. Further validation of the research results is needed in a multicenter prospective cohort, and an in-depth exploration of the interaction mechanism between neutrophils and apoA1 in anti-NMDAR encephalitis is needed to provide a theoretical basis for the development of novel therapeutic strategies targeting neuroinflammation.

## Data Availability

The original contributions presented in the study are included in the article/Supplementary material, further inquiries can be directed to the corresponding author.

## Supplementary material

SUPPLEMENTARY FIGURE 1 Scatter plots of correlations between NAR with initial mRS score **(a)** and CRP **(b)**. NAR, neutrophil-to-apolipoprotein A1 ratio; mRS, modified Rankin scale; CRP, C-reactive protein. **p* < 0.05.
