# Supplementary figures and images for: Neutrophil-to-apolipoprotein A1 ratio as a novel biomarker for prognosis in anti-NMDAR encephalitis: a retrospective cohort analysis
Source: Front Neurol. 2026 Jan 28;17:1725493. doi: 10.3389/fneur.2026.1725493 (PMC12890673; doi:10.3389/fneur.2026.1725493)

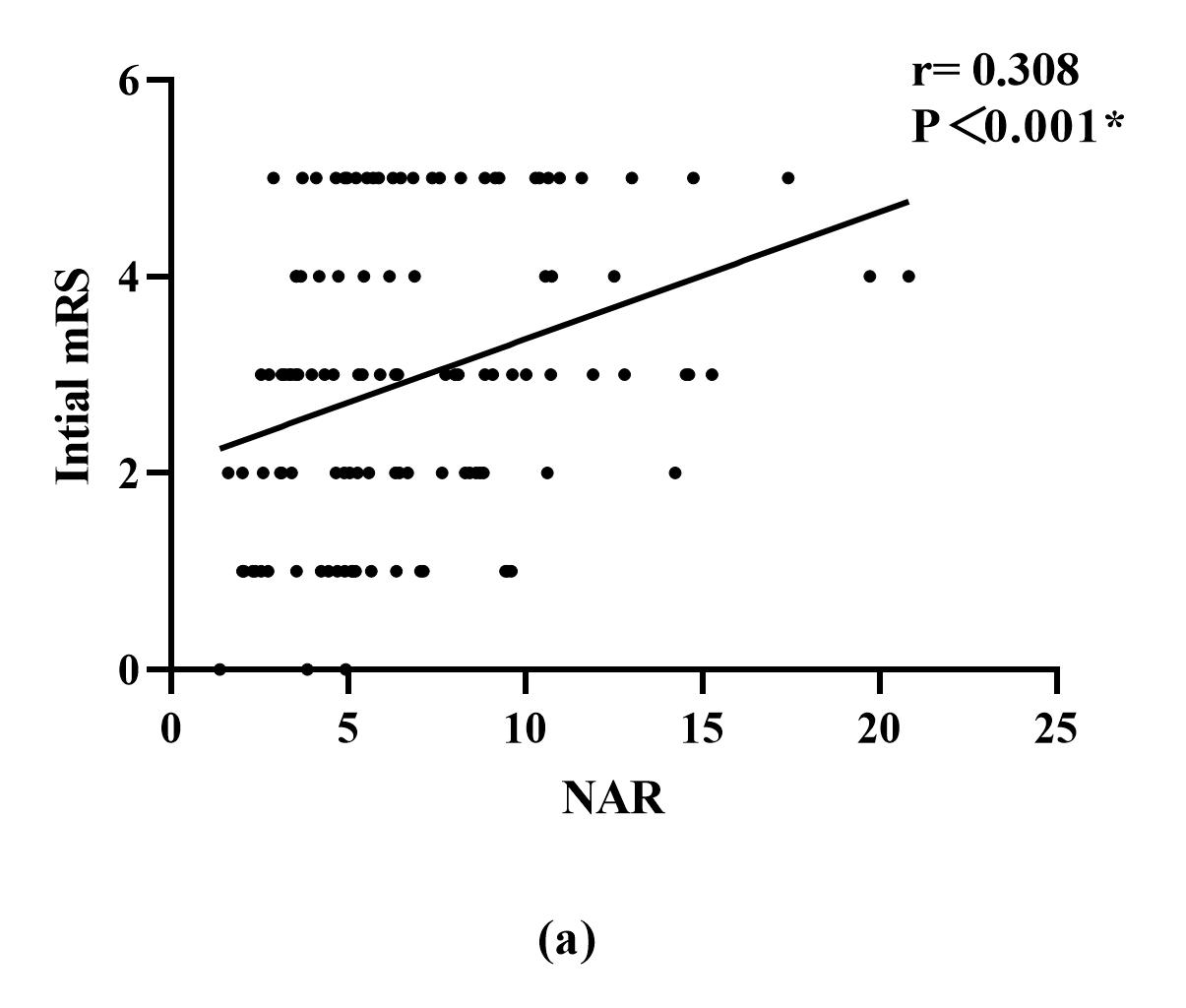

Supplement: SUPPLEMENTARY FIGURE 1 — Scatter plots of correlations between NAR with initial mRS score (a) and CRP (b). NAR, neutrophil-to-apolipoprotein A1 ratio; mRS, modified Rankin scale; CRP, C-reactive protein. *p < 0.05. [file Image_1.jpeg]

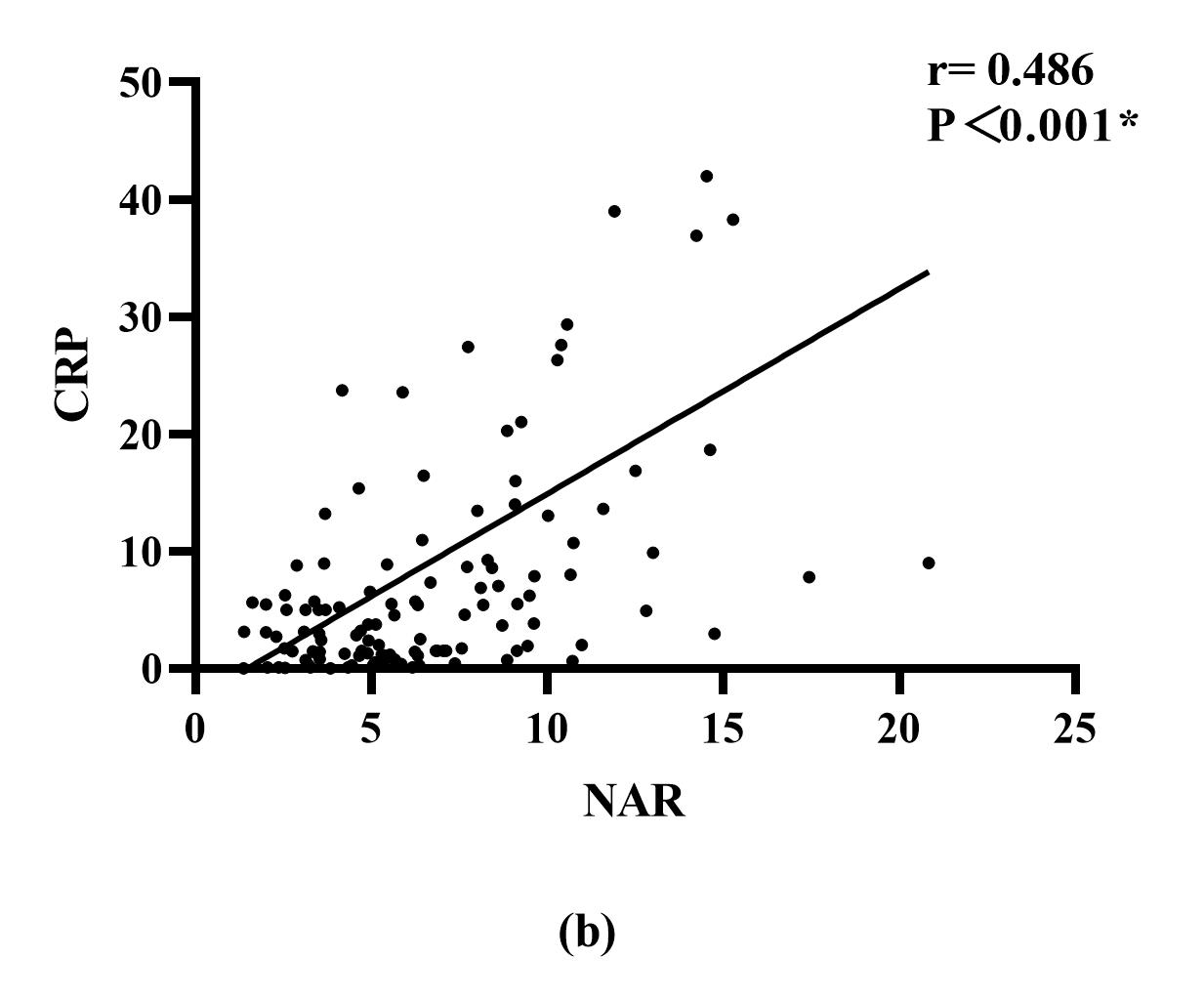

Supplement: Supplementary file 2 [file Image_2.jpeg]
