# Supplementary material for: Neutrophil-to-apolipoprotein A1 ratio as a novel biomarker for prognosis in anti-NMDAR encephalitis: a retrospective cohort analysis
Source: Front Neurol. 2026 Jan 28;17:1725493. doi: 10.3389/fneur.2026.1725493 (PMC12890673; doi:10.3389/fneur.2026.1725493)
Supplement: Supplementary file 3 [file Table_1.docx]

**Supplementary Table 1: List of excluded anti-inflammatory medications**

| ****Medication Category^a, b^**** | ****Generic Name**** |
| --- | --- |
| Non-steroidal anti-inflammatory drugs (NSAIDs) | All systemically administered NSAIDs such as ibuprofen, naproxen, diclofenac, celecoxib, and etoricoxib |
| Systemic corticosteroids | Oral or injectable corticosteroids such as prednisone, methylprednisolone, dexamethasone |
| Other agents with explicit systemic anti-inflammatory effects | Medications like colchicine when used for treating acute gout |

Supplementary information:

^a^Anti-infective medications: In this study, anti-inflammatory medications specifically refer to drugs whose primary pharmacological action was direct anti-inflammatory activity. Routine anti-infective agents (such as antibiotics, antivirals) were not included as direct anti-inflammatory medications. However, to rigorously control for confounding, patients were excluded if they had a clear, active bacterial/viral infection at admission or were undergoing antimicrobial therapy for such an infection, as their baseline inflammatory status (NAR, CRP) could be substantially confounded by the infectious process.

^b^Aspirin: Long-term, low-dose aspirin (typically 75-100 mg/day) for cardiovascular prophylaxis was not part of the exclusion criterion, given its minimal anti-inflammatory effect at this dosage and the potential risk associated with its abrupt discontinuation.

**Supplementary Table 2 Correlation analysis of clinical parameters and disease severity**

| **Factor** | **r** | ***P*** |
| --- | --- | --- |
| Neutrophil, ×10^9^/L | 0.329 | ＜0.001* |
| TC, mmol/L | -0.033 | 0.716 |
| TG, mmol/L | 0.038 | 0.673 |
| HDL, mmol/L | -0.037 | 0.683 |
| LDL, mmol/L | -0.022 | 0.809 |
| Apo-A1, g/L | -0.164 | 0.067 |
| Apo-B, g/L | 0.102 | 0.260 |
| CRP, mg/L | 0.035 | 0.752 |
| NAR | 0.308 | ＜0.001* |

TC, total cholesterol; TG, triglycerides; HDL, high density lipoprotein; LDL, low density lipoprotein; Apo-A1, apolipoprotein A1; Apo-B, apolipoprotein B; CRP, C-reactive protein; NAR, neutrophil-to-apolipoprotein A1 ratio. **P*< 0.05.

**Supplementary Table 3 Correlation analysis of clinical parameters and CRP**

|  | **r** | ***P*** |
| --- | --- | --- |
| Neutrophil, ×10^9^/L | 0.474 | ＜0.001* |
| TC, mmol/L | -0.095 | 0.291 |
| TG, mmol/L | 0.025 | 0.780 |
| HDL, mmol/L | -0.143 | 0.110 |
| LDL, mmol/L | -0.066 | 0.463 |
| Apo-A1, g/L | -0.172 | 0.056 |
| Apo-B, g/L | 0.012 | 0.891 |
| NAR | 0.486 | ＜0.001* |

TC, total cholesterol; TG, triglycerides; HDL, high density lipoprotein; LDL, low density lipoprotein; Apo-A1, apolipoprotein A1; Apo-B, apolipoprotein B; CRP, C-reactive protein; NAR, neutrophil-to-apolipoprotein A1 ratio. **P*< 0.05.

**Supplementary Table 4** Sensitivity analysis of poor outcome in patients excluded autoimmune disease

| **Factor** | **Model I ^a^** | | **Model II ^b^** | |
| --- | --- | --- | --- | --- |
|  | **OR (95% CI)** | ***P*** | **OR (95% CI)** | ***P*** |
| NAR | 1.24(1.09-1.42) | 0.001* | 1.19(1.02-1.38) | 0.025* |

OR, odds ratio; CI, confidence interval; NAR, neutrophil-to-apolipoprotein A1 ratio.

a Model I was adjusted for age, gender and initial mRS; b Model II was adjusted for age, gender, initial mRS, tumor, CRP, and lymphocytes.

*P < 0.05.

**Supplementary Table 5 Sensitivity analysis of relapse in patients excluded autoimmune disease**

| **Factor** | **Model I ^a^** | | **Model II ^b^** | |
| --- | --- | --- | --- | --- |
|  | **HR (95% CI)** | ***P*** | **HR (95% CI)** | ***P*** |
| NAR | 1.12 (1.02-1.23) | 0.013* | 1.12 (1.01-1.23) | 0.032* |

HR, hazard ratio; CI, confidence interval; NAR, neutrophil-to-apolipoprotein A1 ratio.

a Model I was adjusted for age, gender and initial mRS; b Model II was adjusted for age, gender, initial mRS, tumor, and CRP.

*P < 0.05.

**Supplementary table 6 Stratification analysis of poor prognosis in patients with anti- NMDAR encephalitis**

| **Variables** | **N (%)** | **Poor outcome** | | **Relapse** | |
| --- | --- | --- | --- | --- | --- |
|  |  | **OR (95% CI)** | ***P* for interaction** | **HR (95% CI)** | ***P* for interaction** |
| Female | 66 (52.8) | 1.17(0.90-1.52) | 0.246 | 1.04 (0.87-1.24) | 0.665 |
| Male | 59 (47.1) |  |  |  |  |

anti-NMDAR, anti-N-methyl-d-aspartate receptor; OR, odds ratio; CI, confidence interval; HR, hazard ratio.
